# Five Cases of Non-Hodgkin B-Cell Lymphoma of the Ovary

**DOI:** 10.1155/2014/392758

**Published:** 2014-01-22

**Authors:** Taylan Senol, Emek Doger, Ilker Kahramanoglu, Ayfer Geduk, Emre Kole, Izzet Yucesoy, Eray Caliskan

**Affiliations:** ^1^Department of Obstetrics and Gynecology, Kocaeli University Medical Faculty, Kocaeli, Turkey; ^2^Suleymaniye Gynaecologic and Obstetrics Training and Research Hospital, Department of Gynecology, Istanbul, Turkey; ^3^Department of Hematology/Oncology, Kocaeli University Medical Faculty, Kocaeli, Turkey

## Abstract

The involvement of the ovary in lymphomatous process is rare. Such an involvement may occur in 2 ways, primary or secondary. We report 5 cases of ovarian non-Hodgkin's lymphoma, with 3 of which primarily arising in the ovaries. Ovarian lymphoma can mimic more frequently occurring tumors including advanced epithelial carcinoma and radical surgery may be performed instead of a biopsy. The immunophenotypic and clinicopathologic features exhibited in this small series are described to call attention to early diagnosis and treatment of ovarian lymphoma. All patients were diagnosed as having DLBCL after ovary biopsy. Different treatment modalities were used and prognosis of the patients was reported.

## 1. Introduction

Involvement of the ovary by malignant lymphoma, particularly Non-Hodgkin's lymphoma (NHL), is a well-known manifestation of disseminated lymphoma with a frequency of 7% to 26% [1]. However, presumably primary ovarian Non-Hodgkin's lymphoma (PONHL) is rare and accounts for 0.5% of NHL and 1.5% of ovarian tumors [2]. Diffuse large B-cell type accounts for about 20% of PONHL [3]. All of the cases presented in this report were diffuse large B-cell lymphoma. Primary ovarian lymphoma (POL) may be misdiagnosed as epithelial ovarian malignancy. This report describes 5 cases of ovarian NHL, with 3 of which primarily arising in the ovaries. The immunophenotypic and clinicopathologic features exhibited in this small series are described to call attention to early diagnosis and treatment of ovarian lymphoma.

## 2. Case 1

**Figure 1:**
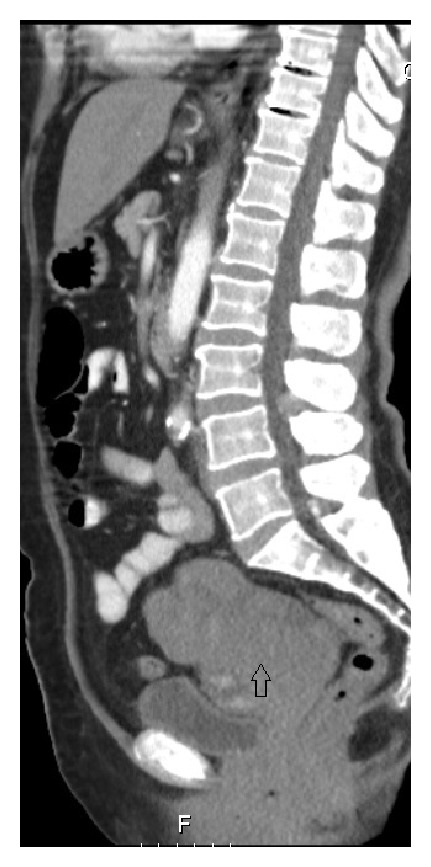
A CT scan showing a large mass of the left ovary.

A 65-year-old, vaginally grand multiparous woman presented to our clinic with symptoms of night sweats and fatigue for a year. She reported 21 kilogram weight loss over the past year. She had no significant medical or family history and she had used no medications. Physical examination revealed a large, palpable, nontender mass with restricted mobility in both lower quadrants up to the level of the umbilicus. A low platelet count was detected, a level of 114 × 10³ *μ*L (the reference level: 142 × 10³ *μ*L). Serum CA 125 and lactate dehydrogenase (LDH) were raised (541 U/mL and 821 IU/L, resp.), but CA 19-9 and CEA were within normal limits. Transabdominal ultrasonography demonstrated a complex adnexal mass, measuring 12.5 × 11 cm with solid areas and internal echoes. A computer tomography (CT) scan revealed an 11 × 9 × 7 cm, solid, left ovarian mass with ascites (Figure 1). The patient underwent surgery with the presumed diagnosis of an ovarian malignancy. At laparotomy, about 2 liters of gelatinous floating material was scooped out of the peritoneal cavity. A 20 × 15 cm pelvic mass, originating from the left adnexal area, adherent to descending colon was found. No definite metastatic lesion was observed. The mass was removed completely, the left ureter was identified, intraoperative frozen section was performed, and a poorly differentiated hematological malignancy was diagnosed. The surgery included hysterectomy and bilateral salpingooophorectomy along with omentectomy, pelvic and inguinal lymph node sampling, appendectomy, peritoneal cytology, and peritoneal biopsy.

**Figure 2:**
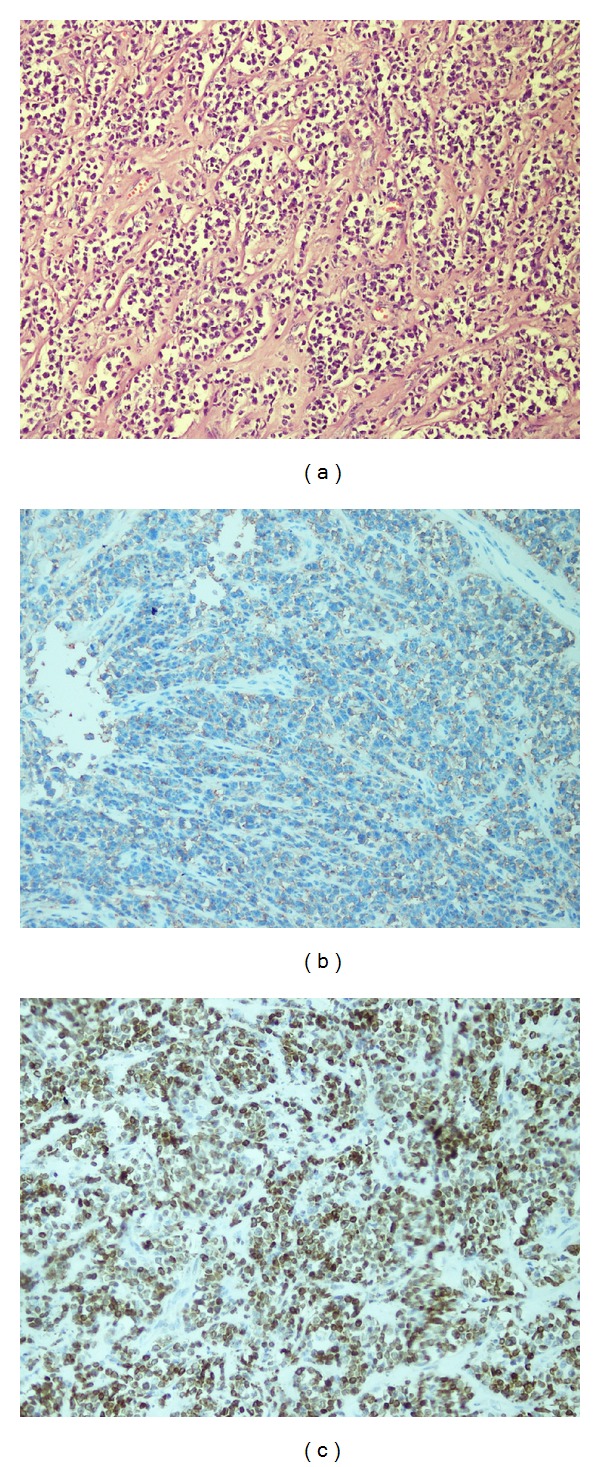
Diffuse infiltration of malignant lymphoid cells (H&E ×100) (a) stained with CD20 (b) and bcl-2 (c) (immunohistochemistry ×40 and ×100, resp.).

Immunohistochemical evaluation showed strong staining for CD20 and bcl-2 (Figure 2). These same cells were negative for pan-cytokeratin, S-100, EMA, CD30, HMB-45, and inhibin. The diagnosis of diffuse large B-cell lymphoma was made. The patient was assessed as stage I BE according to the Ann Arbor system. Bone marrow examination could not be performed due to her poor general condition and chemotherapy was not started. The patient died because of disseminated intravascular coagulation on the postoperative fourteenth day.

## 3. Case 2

A 61-year-old, grand multiparous woman presented to our clinic with a history of painless abdominal swelling of 3 months. She had a history of hypertension and asthma diagnosed five years ago. Upon examination, a large abdominopelvic mass with restricted mobility was detected. Laboratory studies showed elevated levels of CA125 and LDH, 312 U/mL and 417 IU/L, respectively. Other laboratory tests, including CA 19-9 and CEA, were within normal limits. Ultrasonography showed a 15 × 9 cm, complex right adnexal mass with solid and cystic components. A CT scan revealed a 21 × 13 × 8 cm, solid, right ovarian mass with ascites. There was no significant lymphadenopathy or distant metastasis. The patient underwent laparotomy with presumed diagnosis of ovarian malignancy and solid right ovarian mass measuring 20 × 12 cm was found and removed. Intraoperative frozen section evaluation revealed a poorly differentiated epithelial tumor of ovary. The surgery included hysterectomy and bilateral salpingooophorectomy along with omentectomy, pelvic and inguinal lymph node sampling, appendectomy, peritoneal cytology, and peritoneal biopsy. Immunohistochemical evaluation showed strong staining for CD20, CD45, and Ki-67 and weak straining for CD3. These same cells were negative for cytokeratin and epithelial membrane antigen. A diagnosis of stage I BE (Ann Arbor staging) non-Hodgkin's lymphoma (diffuse large B-cell lymphoma) was made. Bone marrow biopsy showed no evidence of malignant cells. The patient was treated with 8 cycles of rituximab, cyclophosphamide, hydroxydaunorubicin, and Oncovin (R-CHOP) chemotherapy. Followup to the 20th month after the surgery showed no evidence of recurrence. The patient died from pneumonia at the 20th postoperative month.

## 4. Case 3

A 53-year-old grand multiparous woman presented with complaints of frequent urination, night sweats, progressing abdominal swelling, and increasing pain in the abdomen of 4-month duration. She had no significant medical or family history and she had used no medications. Physical examination revealed a tender mass of 20-week size in the lower abdomen. Laboratory studies showed elevated levels of CA125 and LDH, 648 U/mL and 454 IU/L, respectively. Other laboratory tests, including CA 19-9 and CEA, were within normal limits. Ultrasonography showed a large amount of free fluid in the cul-de-sac, a left adnexal solid mass measuring 10 × 6 cm, and a right adnexal solid mass measuring 6 × 5 cm, and those were confirmed on a CT scan. Exploratory laparotomy revealed a 10 cm right-sided and 8 cm left-sided solid masses and 15 cm retroperitoneal mass localized in front of the aorta and below the kidneys. At the time of the surgery, 3 L of ascites was evacuated. There were multiple implants ranging from 1 cm to 5 cm on the surface of peritoneum. Frozen section of the resected masses demonstrated a poorly differentiated malignancy. A total hysterectomy and bilateral salpingooopherectomy with omentectomy, lymph node sampling, appendectomy, peritoneal cytology, and peritoneal biopsy were performed. Immunohistochemical evaluation showed strong straining for CD20. These same cells were negative for pan-cytokeratin, S-100, EMA, CD30, CD10, CD5, HMB-45, and inhibin. Bone marrow biopsy showed no evidence of malignant cells. The diagnosis of diffuse large B-cell lymphoma was made and stage was assessed as IV BE according to the Ann Arbor system. After operation, MRI scans showed para-aortic and paracaval bulky lymph nodes. A chemotherapy protocol comprising 6 cycles of R-CHOP regimen was administered to the patient. Radiation therapy was also given for retroperitoneal mass. Followup to 6 months after surgery showed no evidence of recurrence.

## 5. Case 4

A 52-year-old, grand multiparous woman presented to our clinic with symptoms of fatigue and progressive abdominal swelling for 6 months. She had no significant medical or family history. A tender mass of 20-week size from the pelvis to the umbilicus was found on physical examination. CT scan revealed a large 16 × 12 × 10 cm solid right ovarian mass extending to the retroperitoneum with ascites. Serum CA125 was elevated (41 U/mL). Other laboratory tests including CA19-9, CEA, and LDH were within normal limits. On minilaparotomy, wedge of the mass was taken for histopathology examination. On immunohistochemical evaluation, CD20 showed strong staining, while CD5, CD3, and bcl-2 were negative. Histopathology of the ovarian tumour was reported to be consistent with diffuse large B-cell lymphoma. Bone marrow biopsy showed no evidence of malignant cells. The patient was assessed as stage I BE according to the Ann Arbor system. A chemotherapy protocol comprising 8 cycles of R-CHOP regimen was administered to the patient. Followup to 12 months after chemotherapy showed no evidence of recurrence.

## 6. Case 5

**Figure 3:**
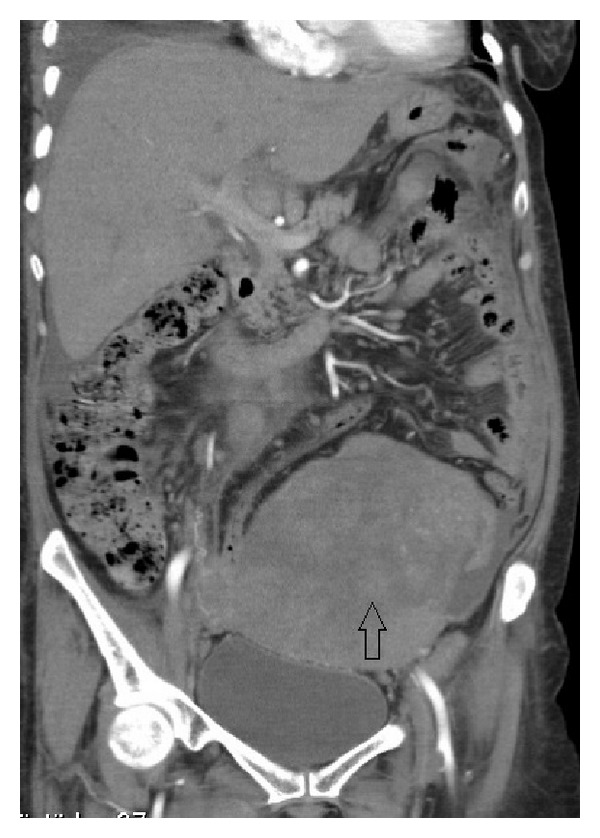
CT scan revealed a large mass of the left ovary with pelvic tissue density.

A 57-year-old grand multiparous woman presented to our clinic with a history of abdominal swelling, night sweats, and fatigue of 2 months. She had no significant medical or family history. Upon examination, a tender mass of 24-week size with restricted mobility was detected. CT scan revealed a 16 × 15 × 10 cm left ovarian mass with ascites (Figure 3). Laboratory studies showed elevated levels of CA125 and LDH, 220 U/mL and 2012 IU/L, respectively. A biopsy of the mass was performed. Immunohistochemical evaluation showed strong straining for CD20 but was negative for CD5 and CD10. The diagnosis of stage I BE and diffuse large B-cell lymphoma was made. Bone marrow biopsy showed no evidence of malignant cells. Chemotherapy could not be started because of her poor general condition and the patient died in a month.

## 7. Discussion

Ovarian involvement by malignant lymphoma is well recognized and is seen at autopsy with a frequency of 7%–26% [1]. However, POL as the initial manifestation is an uncommon entity, accounting for 0.5% of all non-Hodgkin's lymphoma and 1.5% of all ovarian neoplasms [2]. POL can mimic more frequently occurring tumors including advanced epithelial carcinoma as in cases 1, 2, and 3, and, therefore, the correct diagnosis and optimal treatment would have been missed without histopathological examination [4]. These three patients underwent radical surgery with the presumed diagnosis of an epithelial ovarian malignancy, whereas right diagnosis could allow optimal treatment, which is chemotherapy, instead of radical surgery.

Lymphomas of the ovary can occur at any age, but most often in women over the age of 40 (mean, 47 years old in 8 presented cases) [5]. Our patients ranged in age from 52 to 65 years (mean 57 years) (Table 1). Lymphomas of the ovary may have varied presentations such as abdominal pain, pelvic mass, and ascites. Fever, night sweats, fatigue, or weight loss (B symptoms) was noted in 10%–33% of the patients [2, 3]. In our study, 4 of 5 cases presented with an abdominal mass. All of the patients had B symptoms of NHL.

It is difficult to determine whether ovarian lymphomas are primary or secondary. There has been much controversy in the histogenesis of POL. It is demonstrated that normal ovaries contain lymphocytes of B-cell and T-cell lineage within cortical granulomas and rare lymphocytes are present throughout the ovarian stroma and within ovarian follicles and corpora lutea, which can be the source of NHL [1]. Fox et al. proposed the following criteria for the diagnosis of PONHL [6]. (1) At the time of diagnosis, the lymphoma is clinically confined to the ovary and a complete investigation fails to reveal evidence of lymphoma elsewhere. However, an ovarian lymphoma can still be considered as primary if it has spread to immediately adjacent lymph nodes or if it has directly spread to infiltrate immediately adjacent structures. (2) The peripheral blood and bone marrow should not contain any abnormal cells. (3) If further lymphomatous lesions occur at sites remote from the ovary, then at least several months should have elapsed between the appearance of the ovarian and extraovarian lesions. When these criteria are applied, cases 2, 4, and 5 in our report can be diagnosed as PONHL. Although bone marrow biopsy could not be performed because of the patient's poor general condition in case 1, the lymphoma was confined to the ovary as seen on MRI and in surgery. So, lymphoma in case 1 also can be considered as primary ovarian. Diffuse large B-cell lymphoma is the most common subtype of NHL and appears to be the most common type of primary ovarian [5]. All of cases in this report had diffuse large B-cell lymphoma and immunohistochemical evaluation showed strong straining for CD20 for all of them. In case 2, tumour cells were also positive for CD 45 and Ki-67. Patients with ovarian lymphomas are treated with chemotherapy. Radiotherapy is optional [7]. The protocol for chemotherapy used in diffuse large B-cell histology is the standard R-CHOP regimen. Rituximab plays an essential role in treatment of CD20-positive B-cell lymphoma [8]. PONHLs have a poor outcome with a range from 0% to 36% expected to survive for less than three years [2, 9].

In conclusion, prognosis of ovarian lymphomas is often poorer than nodal lymphomas because of delayed or inaccurate diagnosis. The best treatment option seems to be chemotherapy. Physicians should be aware of this rare presentation to avoid radical surgery, which is unnecessary.

## Figures and Tables

**Table 1:** Details of 5 cases of ovarian NHL, presented in this report.

Patient number	Age	Presentation	Size and side	Immunophenotype	Histologic type	Ann Arbor stage	Therapy	Follow up
1	65	21 kg weight loss, night sweats, fatigue	20 × 15 cm left ovarian mass	CD20 (+), bcl-2 (+) pan-cytokeratin (−) S-100 (−) EMA (−) CD30 (−) HMB-45 (−) inhibin (−)	DLBCL	I BE	Sx	Developed multiorgan failure; died of disease 1 month after operation
2	61	Abdominal swelling, fatigue	20 × 12 cm right ovarian mass	CD20 (+) CD45 (+) bcl-2 (+) Ki-67 (+) CD3 (−)	DLBCL	I BE	Sx, CT	Died from pneumonia at the 20th postoperative month
3	53	Abdominal swelling, night sweats, frequent urination, increasing pain in the abdomen	10 × 8 cm right ovarian mass, 8 × 8 cm left ovarian mass, and 15 × 13 cm retroperitoneal mass in front of the aorta	CD20 (+) bcl-2 (+) pan-cytokeratin (−) S-100 (−) EMA (−) CD30 (−) CD10 (−) CD5 (−) HMB-45 (−) inhibin (−)	DLBCL	IV BE	Sx, CT, RT	6 months after surgery, alive with no evidence of recurrence
4	52	Abdominal swelling, fatigue	16 × 10 cm right ovarian mass	CD20 (+) bcl-2 (+) CD5 (−) CD3 (−)	DLBCL	I BE	CT	12 months after chemotherapy, alive with no evidence of recurrence
5	57	Abdominal swelling, fatigue, night sweats	16 × 10 cm left ovarian mass	CD20 (+) bcl-2 (+) CD5 (−) CD10 (−)	DLBCL	I BE	—	Rapidly developed multiorgan failure; died of disease in a month

DLBCL: diffuse large B-cell lymphoma; Sx: surgery; CT: chemotherapy (rituximab, cyclophosphamide, hydroxydaunorubicin, Oncovin, prednisolone (R-CHOP)); RT: radiotherapy.

F-FDG PET/CT: f-18-fluorodeoxyglucose positron emission/computerized tomography.
